# Genome-Wide Analyses of Thaumatin-like Protein Family Genes Reveal the Involvement in the Response to Low-Temperature Stress in *Ammopiptanthus nanus*

**DOI:** 10.3390/ijms24032209

**Published:** 2023-01-22

**Authors:** Qi Liu, Xiangyu Sui, Ying Wang, Ming Zhu, Yijun Zhou, Fei Gao

**Affiliations:** 1 Key Laboratory of Mass Spectrometry Imaging and Metabolomics, Minzu University of China, National Ethnic Affairs Commission, Beijing 100081, China; 2 Key Laboratory of Ecology and Environment in Minority Areas, Minzu University of China, National Ethnic Affairs Commission, Beijing 100081, China; 3 College of Life and Environmental Sciences, Minzu University of China, Beijing 100081, China

**Keywords:** thaumatin-like protein, *Ammopiptanthus nanus*, osmotic stress, low temperature, gene family

## Abstract

Thaumatin-like proteins (TLPs), a family of proteins with high sequence similarity to thaumatin, are shown to be involved in plant defense, and are thus classified into the pathogenesis related protein family 5. *Ammopiptanthus nanus* is a rare evergreen broad-leaved shrub distributed in the temperate zone of Central Asia, which has a high tolerance to low-temperature stress. To characterize *A. nanus* TLPs and understand their roles in low-temperature response in *A. nanus*, a comprehensive analysis of the structure, evolution, and expression of TLP family proteins was performed. A total of 31 *TLP* genes were detected in the *A. nanus* genome, and they were divided into four groups based on their phylogenetic positions. The majority of the AnTLPs contained the conserved cysteine residues and were predicted to have the typical three-dimensional structure of plant TLPs. The primary modes of gene duplication of the AnTLP family genes were segmental duplication. The promoter regions of most *AnTLP* genes contain multiple cis-acting elements related to environmental stress response. Gene expression analysis based on transcriptome data and fluorescence quantitative PCR analysis revealed that several *AnTLP* genes were involved in cold-stress response. We further showed that a cold-induced *AnTLP* gene, *AnTLP13*, was localized in apoplast, and heterologous expression of the *AnTLP13* in *Escherichia coli* and yeast cells and tobacco leaves enhanced low-temperature stress tolerance when compared with the control cells or seedlings. Our study provided important data for understanding the roles of TLPs in plant response to abiotic stress.

## 1. Introduction

Thaumatin-like proteins (TLPs) are polypeptides composed of approximately 200 amino acid residues, and their sequences are similar to thaumatin. Thaumatin is a sweet-tasting protein, which was originally found in the *Thaumatococcus daniellii* in West African [1]. Plant TLPs are classified as pathogenesis-related protein family 5 (PR5) due to their induced expression under the invasion of pathogens and pests [2]. Most TLP proteins contain a highly conserved motif, G-X-[GF]-X-C-X-T-[GA]-D-C-X(1,2)-G-X-(2,3)-C, a REDDD (arginine, glutamic acid, and three aspartic acid residues) structure, and sixteen or ten conserved cysteine residues, which form eight or five disulfide bonds [2,3,4]. These disulfide bonds help to maintain the three-dimensional structures of TLPs in unfavorable environments with high temperature or low pH. TLPs have been systematically identified in many plant species, such as barley [5], melon [6], watermelon [7], *Vitis vinifera* [8], and bread wheat [9].

TLPs have been shown to be involved in defense systems against various biotic stresses, and many TLP proteins have broad-spectrum antifungal activity [2,10]. Compared with wild-type, rice seedlings overexpressing *TLP* showed higher tolerance to *Rhizoctonia solani* and *Sarocladium oryzae* [11]. *Arabidopsis thaliana* plants overexpressing the *VvTLP29* gene from grape showed stronger resistance to powdery mildew [8]. The TLP protein purified from banana exerts its antifungal activity by inducing fungal cell membrane disorder and cell wall disintegration [12]. Some recent studies showed that TLPs were also involved in plant response to abiotic stress. For example, ectopic expression of a TLP gene from peanut enhances the tolerance of tobacco seedlings to salt and oxidative stress [13]. Compared with the control, *Saccharomyces cerevisiae* overexpressing wheat *TaTLP2* gene exhibited stronger tolerance to cold, heat, osmotic, and salt stress [9].

Plants usually live in ever-changing environments that are often unfavorable for plant growth and development. These unfavorable environmental factors include biotic stresses, such as pests or weeds, and abiotic stresses, including low temperature, drought, and high salinity [14,15]. Low temperature and drought are the main environmental factors affecting plant distribution and crop yield. It is estimated that low-temperature stress may lead to about 40% annual crop yield reduction in temperate regions [16]. Low-temperature stress can induce osmotic and oxidative stress, resulting in reactive oxygen species accumulation, protein denaturation, cell membrane damage, nucleic acid damage, and even plant death [17]. Plants can adapt to environmental stress by activating molecular networks, including signal transduction, stress perception, metabolite production, and expression of stress-tolerance related genes. Stress-tolerance related genes mainly include functional genes that encode proteins protecting cellular components, and regulatory genes that regulate stress responses [18]. At present, many functional genes for protecting cells have been characterized, including heat shock protein (HSP), late embryogenesis-abundant (LEA) protein, antioxidant enzymes, and membrane transporters [19]. Mining novel stress-tolerance related genes from plants in special habitats can provide candidate genes for cultivating crop varieties that can tolerate abiotic stresses.

*Ammopiptanthus nanus* is mainly distributed in Wuqia County, Xinjiang Uygur Autonomous Region, China, and Kyrgyzstan. *A. nanus* is a rare evergreen broad-leaved shrub in temperate desert areas and plays an important role in maintaining the fragile plant ecosystem in dryland of central Asia [20]. *A. nanus* has grown in the harsh desert environment of Central Asia for a long time and has a high tolerance to environmental stresses, including low-temperature stress [21]. The stress-related genes in the *A. nanus* genome might contribute to the high abiotic stress tolerance of *A. nanus*. Some stress-related genes of *A. nanus*, including *AnGoIS1* [22] and *AnVP1* [23], have been isolated and characterized by overexpressing in Arabidopsis plants. Preliminary studies have shown that some *TLP* genes are up-regulated under low-temperature stress, thus it is speculated that *TLP* genes might be involved in the response of plants to low-temperature stress in *A. nanus*. In the present study, the whole-genome identification of the TLP family of *A. nanus* was conducted, and the chromosome distribution, phylogenetic relationships, gene replication events, and expression profiling for the TLP gene family members under low-temperature stress in *A. nanus* were analyzed. The biological function of an *AnTLP* gene was further investigated by being expressed in *E. coli*, yeast, and tobacco seedlings. This study will provide important data for understanding the biological function of TLPs in *A. nanus*.

## 2. Results

### 2.1. Genome-Wide Identification of TLPs in A. nanus

A total of 31 TLPs were identified from the genome of *A. nanus* (Table 1). The pI values of the 31 predicted TLPs range from 4.27 to 9.25. The amino acid length of AnTLPs ranged from 196 (AnTLP7) to 348 (AnTLP21), with a molecular weight of 20.9 kDa to 35.1 kDa, respectively. There were 21 acidic proteins in the AnTLP family, accounting for 74.19% of all AnTLPs. A total of 20 AnTLPs were hydrophilic proteins, accounting for 64.52%. Signal peptides were detected in 24 AnTLPs, with an average length of 26 amino acids. There were two transmembrane domains in AnTLP24 and AnTLP28, and one transmembrane domain in 13 AnTLP proteins. Subcellular localization analysis showed that the majority of AnTLPs were located in apoplast.

**Figure 1:**
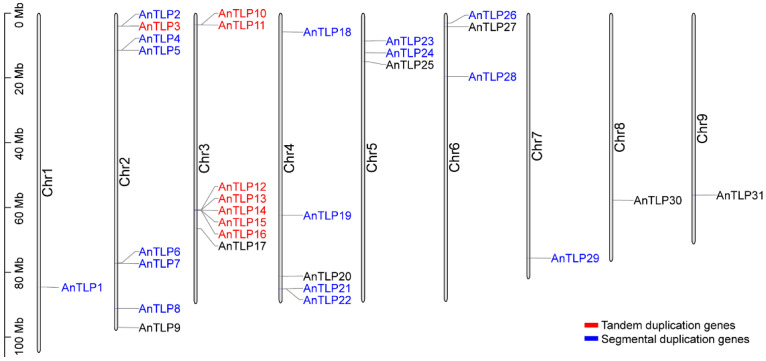
The distribution of *AnTLP* genes in the chromosomes of *A. nanus*. Different colors represented gene replication type. Blue represented fragment replication, and red represented tandem replication.

The 31 *AnTLP* genes were unevenly distributed on all chromosomes of *A. nanus* (Figure 1). There were eight *AnTLP* genes on chromosomes 2 and 3, while only one *AnTLP* gene was found on chromosomes 1, 7, 8, and 9. 

### 2.2. Structural Analysis of TLPs in A. nanus

**Figure 2:**
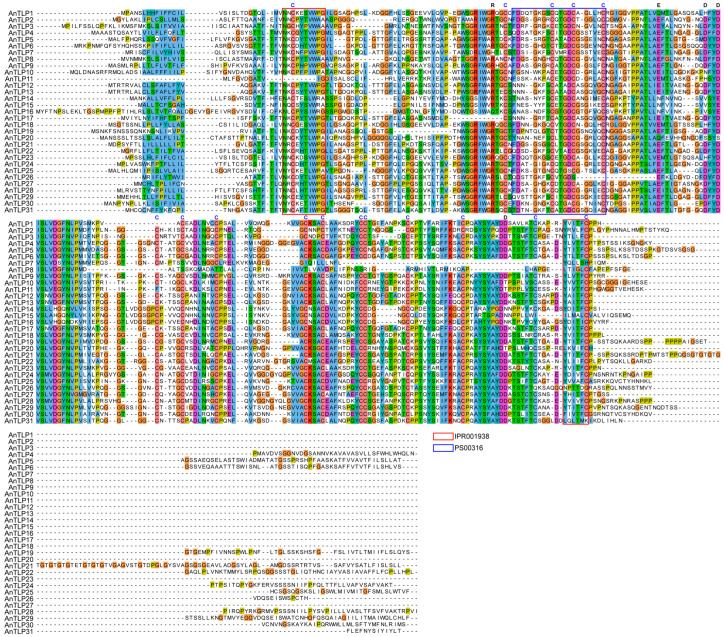
Multiple sequence alignment of TLPs in *A. nanus*. Jalview software was used to visualize the results of multiple sequence alignment, and the color scheme was selected as ‘Clustal’. Conservative domain was used by box (iPR001938, red box; PS00316, blue box). The amino acids in the REDDD motif are highlighted in black letters. The conserved cysteines are highlighted in blue letters.

Amino acid sequences of all the AnTLPs were used to conduct multiple sequence alignment for identification of the conserved domains. As shown in Figure 2, the majority of the AnTLPs possess the typical 16 conserved cysteine residues, while AnTLP8 (EVM0011680) and AnTLP7 (EVM0027347) contain only 6 or 7 cysteine residues, respectively. Most AnTLPs contain the conserved signatures (PS00316, blue box in Figure 2; IPR001938, red boxes in Figure 2), and the conserved REDDD amino acid sequences.

**Figure 3:**
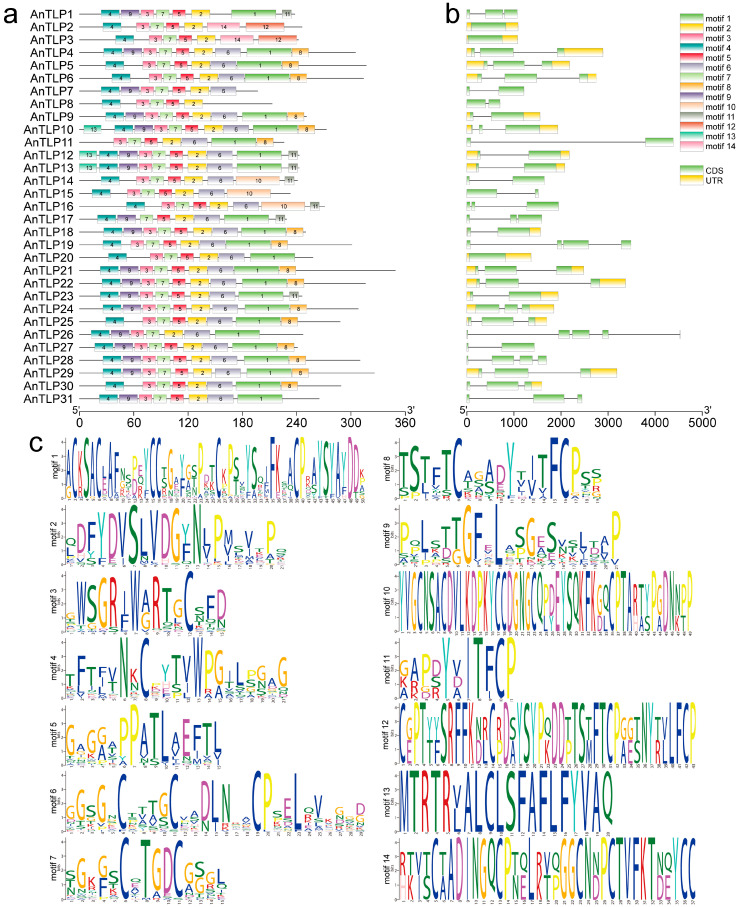
Conserved motif and gene structure of *TLP* gene family in *A. nanus*. (**a**) The conserved motif analysis of the TLP family of *A. nanus*. All motifs were identified using the MEME database. The rectangular box represents the motif, and different colors represent different motifs. The lengths of the proteins and motifs can be estimated using the scale at the bottom. (**b**) The intro–exon structure of the TLP gene family. Black lines represent introns, yellow rectangles represent CDS, and green rectangles represent untranslated regions (UTRs). The size of exons and introns can be estimated using the scale at the bottom. (**c**) Motif sequence logo graph. The relative size of the letters represents their frequency in the sequence. The height of each letter is proportional to the frequency of occurrence of the corresponding base at that position.

We also conducted MEME analysis to identify the protein motif in amino acid sequences of AnTLPs. The majority of the AnTLP proteins contained motif1, motif 2, motif 3, motif 4, motif 5, motif 6, motif 7, motif 8, and motif 9, indicating that these motifs were the core sequences of the conserved domain of the AnTLPs. Some motifs were only detected in one or two AnTLPs. For example, motif 12 and motif 14 were only present in AnTLP2 and AnTLP3 (Figure 3a). All *AnTLP* loci ranged in length from 591 bp to 1047 bp, with the longest gene being AnTLP21 and the shortest being AnTLP7. Most *AnTLP* (28/31, 90.32%) loci contain less than three introns, and 14 *AnTLP* loci have only one or zero intron (Figure 3b).

**Figure 4:**
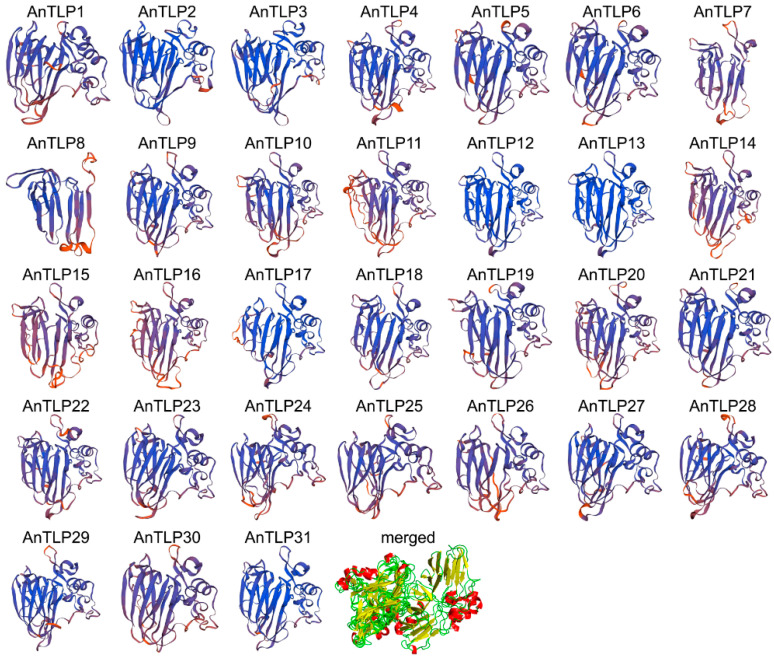
Predicted three-dimensional structures of AnTLPs. The three-dimensional structure prediction was performed in the SWISS-MODEL database. Multiple protein models were combined using PyMol software.

To clarify the differences between AnTLP protein structures, the structure of AnTLP protein was predicted by homology modeling (Figure 4), and the quality of the predicted protein structure was evaluated (Table S1). There were 2–6 α-helix and 10–15 β-sheet and multiple loops in the three-dimensional structure of AnTLP, which was consistent with the typical three-dimensional structure of TLP protein. The identity of AnTLP and template ranged from 36.19% to 75.11%, a value greater than 30%, indicating compliance with homology modelling requirements. Three assessment results showed that the reliability of the predicted three-dimensional structure of AnTLP was high in this present study. AnTLP2, AnTLP3, and AnTLP8 were clearly distinguished from other AnTLP in the merged diagram, indicating that their biological functions might be quite different from other AnTLP.

### 2.3. Phylogenetics, Gene Duplication, and Divergence of TLP Family in A. nanus

**Figure 5:**
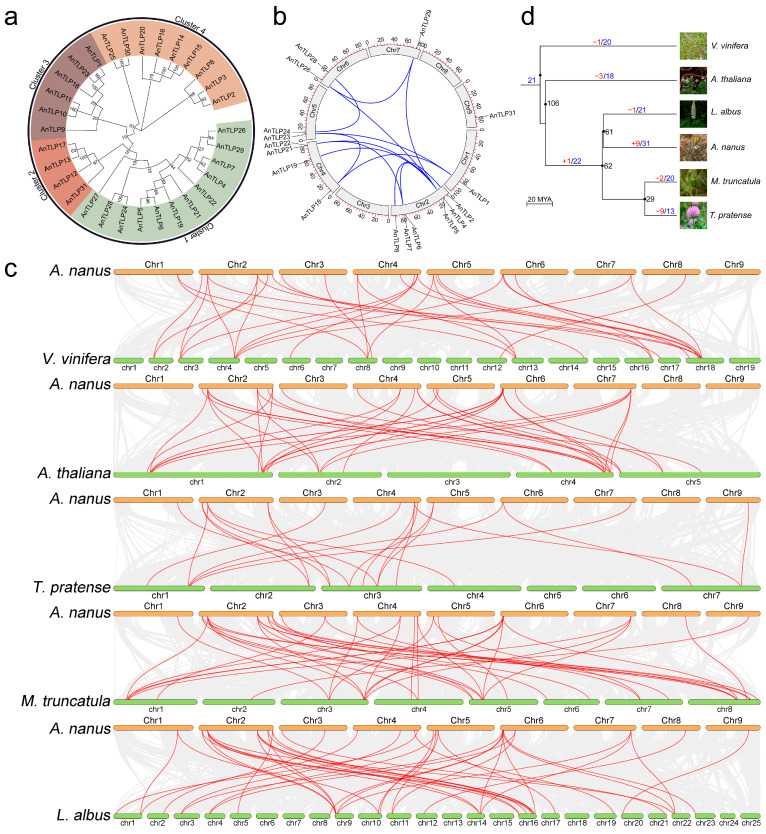
Phylogenetics, gene duplication and divergence of *AnTLP* genes. (**a**) The phylogenetic tree of AnTLP family. Different colors represented different cluster. (**b**) The distribution of segmental duplication genes of *AnTLP* on the chromosome of *A. nanus*. (**c**) Homologous gene pairs between *A. nanus* and *V. vinifera*, *A. thaliana*, *M. truncatula*, *T. pratense*, and *L. albus*, respectively, and the red lines indicate AnTLPs. (**d**) Evolution of TLP gene family in *A. nanus* and five relative species. Black numbers represent the time of species differentiation, red numbers represent the number of orthologous genes of AnTLP lost or increased during evolution, and blue numbers represent the number of orthologous genes in *AnTLP* genes. The gene names represented by red and blue numbers are listed in Table S2. The pictures of *A. nanus*, *V. vinifera*, *A. thaliana*, *M. truncatula*, *T. pratense*, and *L. albus* were obtained from the Plant Photo Bank of China (PPBC).

To analyze the evolutionary relationships of the TLP family members in *A. nanus*, a phylogenetic tree was generated using the amino acid sequences of the predicted AnTLPs (Figure 5a). All AnTLPs were divided into four clusters based on their phylogenetic relationships, with the largest number of AnTLP in Cluster 1 and the smallest number of AnTLP in Cluster 2.

Gene duplication is considered as one of the main driving forces of genome evolution, and segmental duplication and tandem duplication are regarded as two main driving forces for gene family expansion in plants. A total of 16 genes that were generated by segmental duplication and 8 genes that were generated by tandem duplication were identified in the TLP gene family of *A. nanus* (Figure 1 and Figure 5b).

To reveal the evolution of the TLP gene family in *A. nanus*, TLP orthologs in related plant species were identified, and 20, 18, 13, 20, and 21 orthologs of AnTLP were found in *Vitis vinifera*, *A. thaliana*, *Trifolium pratense*, *Medicago truncatula*, and *Lupinus albus*, respectively. Gene collinearity analysis showed that there was a varying degree of collinearity between the TLP gene family of *A. nanus* and the TLP gene family of other plants (Figure 5c). We further reconstructed the evolution relationship between the TLP gene family in *A. nanus* and the other five plant species (Figure 5d).

### 2.4. Positive Selection and Codon Usage Bias Analysis of TLP Gene Family in A. nanus

To investigate the adaptive evolution of the TLP gene family in *A. nanus*, the ratio of non-synonymous mutation (Ka) to synonymous mutation (Ks) of AnTLP was calculated. The Ka/Ks value of the gene for neutral selection was 1, the Ka/Ks value of the gene for negative selection was less than 1, and the Ka/Ks value of the gene for positive selection was higher than 1. All Ka/Ks values for AnTLP gene paralogous pairs were less than 1, indicating that all AnTLPs have undergone purifying selection (Table 2).

**Figure 6:**
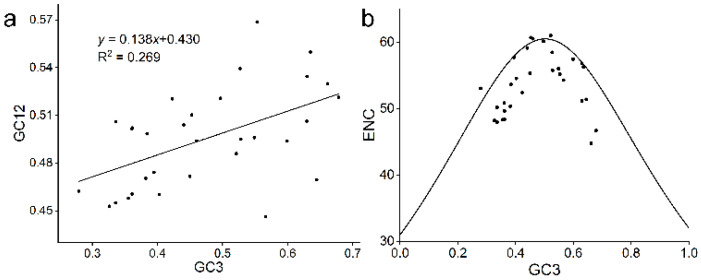
The correlation analysis of GC3, GC12, and ENC in *A. nanus*. (**a**) The correlation analysis of GC3 and GC12. (**b**) The correlation analysis of GC3 and ENC.

Codon usage bias of TLP gene family was analyzed in *A. nanus* to better understand the adaptive evolution of AnTLP. The ENC of AnTLP gene was between 44.78 and 61, the CAI value was between 0.17 and 0.31, and the Fop was between 0.30 and 0.55 (Table 3). The Pearson correlation coefficient for GC12 and GC3s of AnTLP was 0.519, indicating that codon usage bias of AnTLP was affected by mutation pressure (Figure 6a). Most of the points in the association map of ENC and GC3s are distributed near the standard curve, indicating that the codon usage bias of AnTLP was affected not only by mutation pressure, but also by selection pressure (Figure 6b).

### 2.5. Prediction of the Cis-Acting Elements in Promoter Regions of the TLP Genes in A. nanus

**Figure 7:**
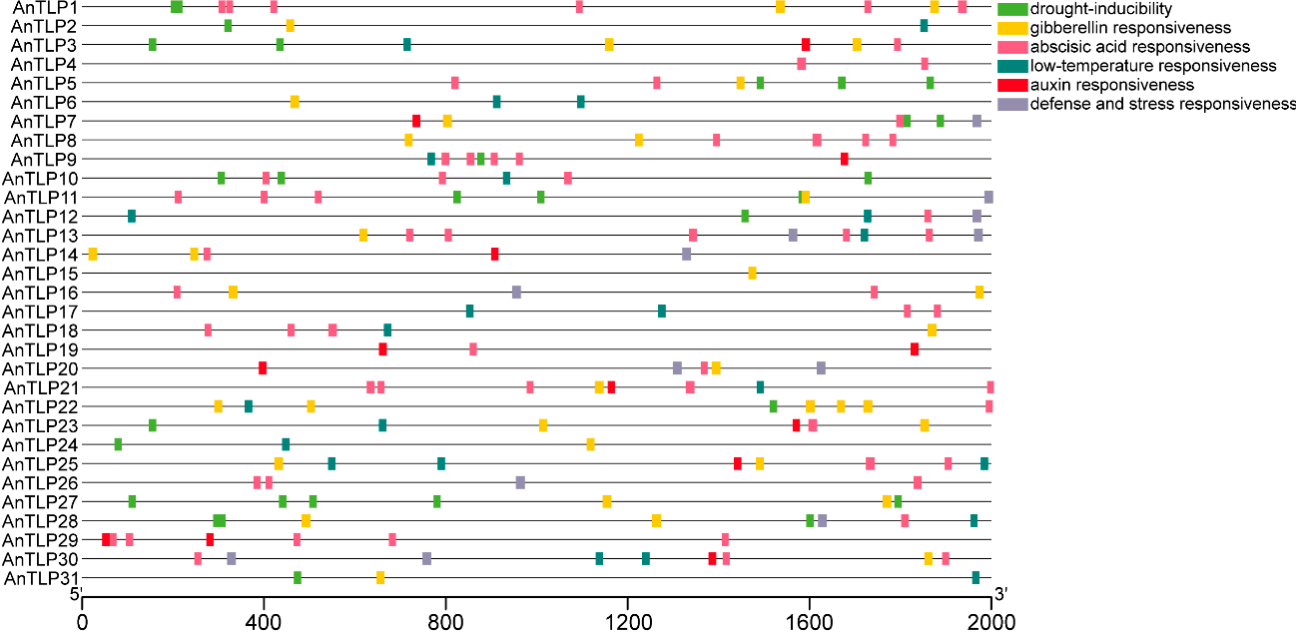
Distribution of the predicted cis-acting elements that were involved in abiotic stress response and hormone response in the promoter region of the *AnTLP* genes in *A. nanus*. Different color blocks represent different types of cis-acting elements. The position of the cis-acting element in the promoter region can be estimated by the scale at the bottom.

To understand the possible biological functions of *AnTLPs*, the cis-acting elements in the promoter regions of *AnTLP* genes were predicted (Figure 7). A total of 17 kinds of cis-acting elements that were involved in abiotic stress response and hormone response were predicted, and these cis-acting elements were involved in plant response to multiple stress signal and hormones, including auxin responsiveness (TGA-box), salicylic acid responsiveness (TCA-element), abscisic acid responsiveness (ABRE), low-temperature responsiveness (LTR), and drought inducibility (MBS). The number of cis-acting elements of the *AnTLP1* and *AnTLP21* genes was the largest, while the number of cis-acting elements of the *AnTLP15* gene was the least. The presence of the LTR element in the promoter regions of 17 *AnTLP* genes suggested that these genes might be involved in the low-temperature stress response in *A. nanus*, and the presence of the MBS element in the promoter regions of 15 *AnTLP* genes suggested that these genes might be involved in the drought stress response in *A. nanus*.

### 2.6. Expression Patterns of A. nanus TLP Genes under Osmotic and Cold Stresses

**Figure 8:**
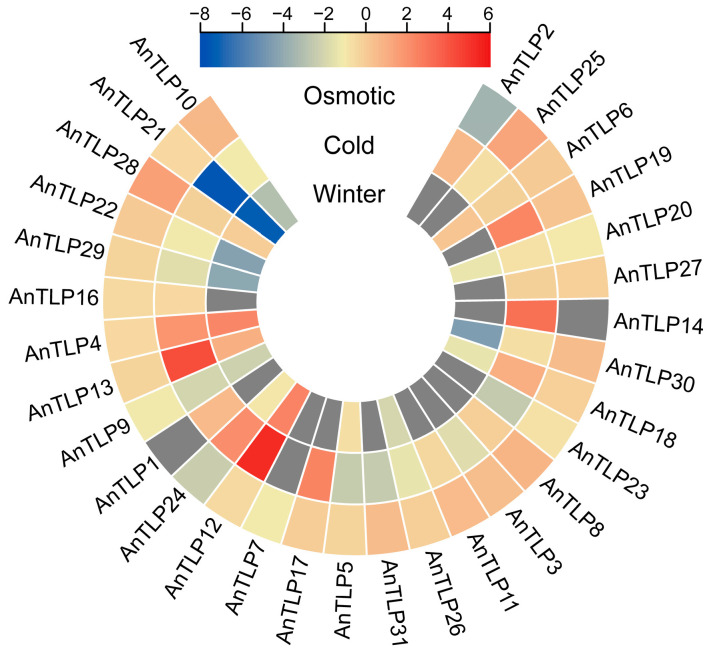
Expression pattern analysis of AnTLP genes based on transcriptome data.

To further evaluate the potential functions of AnTLP genes, especially their roles in osmotic and low-temperature stress responses, the expression patterns of TLP family genes were analyzed using the RNA-seq database and qRT-PCR analysis. The transcriptome data showed that three *AnTLP*, i.e., *AnTLP4*, *AnTLP12*, and *AnTLP13*, were highly expressed in winter, and more *AnTLP* genes were up-regulated under short-term cold stress, including the three highly expressed genes in winter (Figure 8). The transcriptome data revealed that AnTLP25 and AnTLP28 were highly expressed under short-term osmotic stress.

**Figure 9:**
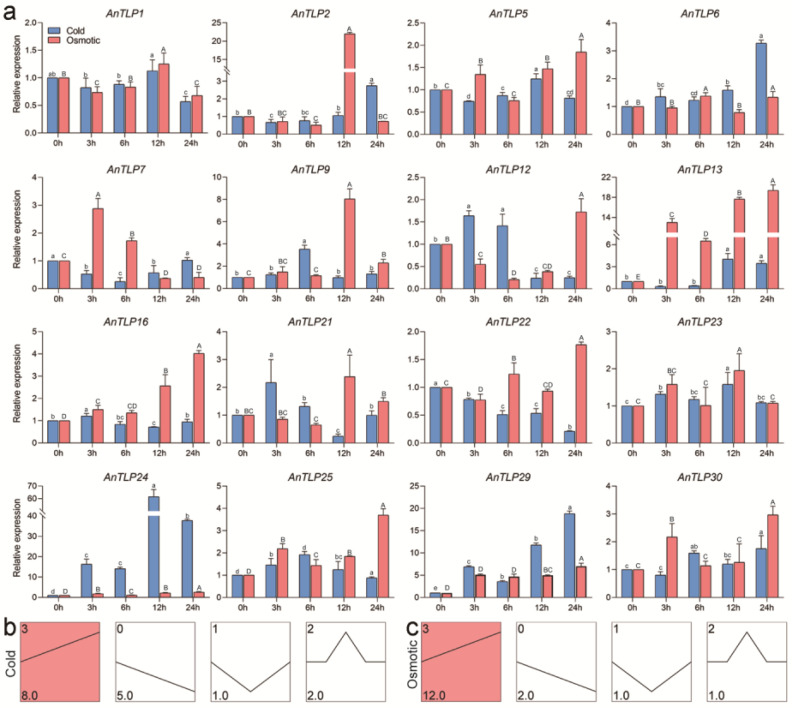
Expression patterns of *AnTLP* genes under cold and osmotic stress. (**a**) Analysis of temporal expression patterns of *AnTLP* genes under cold and osmotic stress; blue represents cold stress and red represents osmotic stress. The *eIF1* gene was used as the internal control gene. The significance between the data of the cold-stress experimental group is marked with lowercase letters, and the significance between the data of the osmotic stress experimental group is marked with uppercase letters. There is no significant difference between the data sharing the same letters; conversely, there are significant differences between the data. (**b**) Trend analysis of *AnTLP* genes expression patterns under cold stress. (**c**) Trend analysis of *AnTLP* genes expression patterns under osmotic stress. The colored segment is a significantly enriched module. The number in the upper left corner of the module is the number of the module, and the number in the lower right corner represents the number of genes belonging to the module.

To further investigate the temporal expression pattern of the *AnTLP*, qRT-PCR analyses were performed for 16 *AnTLP* genes (Figure 9a). By series test of cluster analysis, AnTLP genes can be classified into four categories based on expression patterns under cold stress and osmotic stress (Figure 9b,c). Under cold stress, the expression levels of eight *AnTLP* genes increased gradually, including AnTLP24 and AnTLP29, while the expression of five *AnTLP* genes decreased gradually, including AnTLP16 and AnTLP22. Under osmotic stress, the expression of 12 *AnTLP* genes increased gradually, including AnTLP14 and AnTLP29, and the expression levels of two other *AnTLP* genes decreased gradually, including *AnTLP1* and *AnTLP7*.

### 2.7. Overexpression of AnTLP13 Gene Enhanced the Tolerance to Low-Temperature Stress in E. coli and Yeast

**Figure 10:**
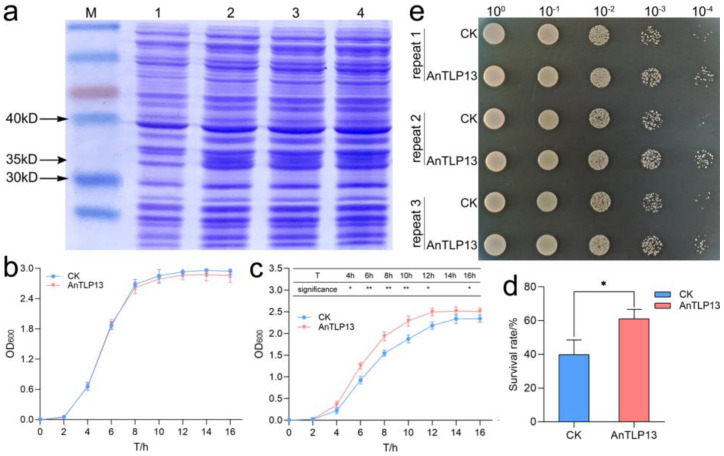
Overexpression of the *AnTLP13* gene enhanced the tolerance to low temperature in *E. coli* and yeast. (**a**) Gel electrophoresis of AnTLP13. M in the figure represents the protein marker, and 1 represents the CK group, 2–4 represent the AnTLP13 group. (**b**) Growth curves of *E. coli* under normal conditions (37 °C). (**c**) Growth curves of *E. coli* under low-temperature stress (28 °C). (**d**) Survival rate of *E. coli* under repeated freeze–thaw stress. * and ** indicate *p* < 0.05 and *p* < 0.01, respectively. (**e**) Overexpression of the *AnTLP13* gene enhanced the tolerance to repeated freeze–thaw stress in yeast.

*E. coli* and yeast growth assay was performed to evaluate the effect of the *AnTLP13* gene on the tolerance of *E. coli* and yeast to low-temperature stress. The AnTLP13 protein bands were found in SDS-PAGE gel, indicating that the *AnTLP13* gene was successfully expressed in *E. coli* cells (Figure 10a). The growth curve of *E. coli* transformed with empty plasmid (CK) was almost coincident with that of *E. coli* overexpressing *AnTLP13*, indicating that overexpression of AnTLP13 did not affect the growth of *E. coli* under normal growth conditions (Figure 10b). When cultured at 28 °C, the growth rate of *E. coli* slowed down, and overexpression of the *AnTLP13* gene effectively alleviated the inhibitory effect of cold on the growth of *E. coli* cells (Figure 10c). Repeated freeze–thaw treatment resulted in the mass death of *E. coli* cells, and overexpression of the *AnTLP13* gene significantly increased the survival rate of *E. coli* after repeated freeze–thaw treatment (Figure 10d). After repeated freeze–thaw treatment, the survival yeast expressing the *AnTLP13* gene (recorded as AnTLP13) was significantly higher than the yeast transformed with the pYES2 empty plasmid (recorded as CK) (Figure 10e). The above results showed that overexpression of the *AnTLP13* gene enhanced the tolerance to low temperature in both *E. coli* and yeast.

### 2.8. Overexpression of AnTLP13 Gene Enhanced the Tolerance of Tobacco to Freezing Stress

**Figure 11:**
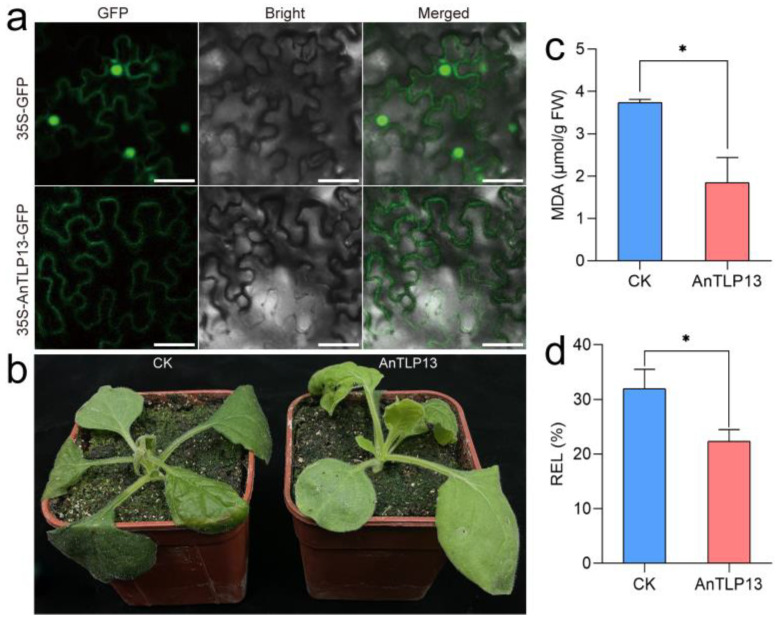
Overexpression of the *AnTLP13* gene enhanced the tolerance of tobacco seedlings to freezing stress. (**a**) Subcellular localization analysis of AnTLP13 during transient expression in tobacco. Bars = 50 μm. (**b**) Tobacco seedlings of CK and AnTLP13 group were transferred to −4 °C for freezing-stress treatment for 12 h. (**c**) The MDA content of tobacco leaves after freezing-stress treatment. (**d**) The values of electrolyte leakage (REL) of tobacco leaves after freezing-stress treatment. ‘*’ indicated *p* < 0.05.

Tobacco transient transformation assay was used to evaluate the protective effect of AnTLP13 on tobacco cells under freezing stress. The tobacco transformed with pCAMBIA1300 empty plasmid was recorded as the CK group, and the tobacco transformed with AnTLP13 was recorded as the AnTLP13 group. Subcellular localization analysis showed that AnTLP13 protein was localized in the apoplast of plant cells (Figure 11a). After tobacco was cultured at −4 °C for 12 h, tobacco seedlings in CK and AnTLP13 groups wilted, but the wilting degree of plants in the AnTLP13 group was relatively low (Figure 11b). MDA and REL are important indicators to evaluate the damage to cell membranes caused by stress conditions, and the MDA content and REL values in the AnTLP13 group were significantly lower than those in the CK group (Figure 11c,d). These data indicate that the expression of the *AnTLP13* gene enhanced the tolerance of tobacco seedlings to freezing stress.

## 3. Discussion

*A. nanus* is a rare evergreen broad-leaved shrub found in the desert area of Central Asia, and this shrub has higher tolerance to environmental stress, including low-temperature and drought stress. The stress-related genes in the *A. nanus* genome were considered to contribute to its extremely high level of tolerance to abiotic stress. TLPs are a class of pathogenesis-related proteins, and most TLPs are predicted to be localized into apoplast, where most PR proteins exist. TLPs have been shown to be involved in plant defense by acting as antifungal proteins [1,2,4], and recent evidence has suggested that TLPs might also be involved in abiotic stress response and tolerance in plant. Previous studies have indicated that apoplast proteins such as chitinases might be involved in the response to low-temperature and drought stress in *A. nanus* [24]. In the present study, we performed a systematically identification of the TLP family in *A. nanus*, investigated the structure, evolution, and expression profiles of AnTLPs, and analyzed their biological function in abiotic stress response.

At present, TLP genes have been systematically identified in many species, but the number of *TLP* genes in different plant species is different. There are more *TLP* genes in *Populus trichocarpa* [25] and *Zea mays* [26], with 55 and 49 TLP family genes, respectively, while there are fewer TLP genes in *Pinus monticola* [27], with only 6 TLP genes. In the present study, 31 TLP genes were identified from the *A. nanus* genome. Using the same identification method, 32, 25, 49, 23, and 29 TLP genes were identified from *V. vinifera*, *A. thaliana*, *M. truncatula*, *T. pratense*, and *L. albus*. It is noteworthy that the number of TLP genes in *M. truncatula* were significantly higher than those of other plant species. Although *M. truncatula*, *T. pratense*, *L. albus*, and *A. nanus* are all leguminous plants, their TLP gene numbers were obviously different. It was speculated that the TLP gene family of *M. truncatula* might have undergone gene expansion.

The orthologous gene pairs between *A. nanus* and *V. vinifera*, *A. thaliana*, *T. pratense*, *M. truncatula*, and *L. albus* were identified in the present study (Figure 5c). In general, the closer the relation between two species is, the greater the number of orthologous gene pairs can be detected between them [28,29,30]. In this present study, the relation between *L. albus* and *A. nanus* was the closest, and the number of orthologous gene pairs between the two species was also the largest (21 pairs). However, only 13 orthologous *TLP* gene were detected between *A. nanus* and *T. pratense*, which was far less than that of other legumes. This result indicated that, in addition to the genetic relationship among species, other factors also affect the number of orthologous gene pairs of the same gene family among different plant species. The above analysis showed that there were only 23 *TLP* genes in the genome of *T. pratense*, and the number was significantly less than that of other legumes, indicating that the *TLP* gene family may shrink in *T. pratense*. This explains why there were fewer orthologous genes between TLPs in *A. nanus* and *T. pratense*.

The evolutionary history of the *TLP* gene family members of *A. nanus* were reconstructed in the present study based on the collinearity analysis of the *TLP* gene families of *A. nanus* and other related five plant species (Figure 5c). There were 21 orthologous genes of *AnTLP* in the genome of the common ancestor species of *V. vinifera* and *A. thaliana*, three of which might be lost in the process of evolution to *A. thaliana*. The orthologous genes of *AnTLP5* were lost before the speciation of *V. vinifera*. The orthologous gene of *AnTLP7* was formed by means of segmental duplication, when the common ancestor species of *V. vinifera* and *A. thaliana* diverged into the common ancestor species of *A. nanus*, *M. truncatula*, *T. pratense*, and *L. albus*. The orthologous gene of *AnTLP7* was lost when the common ancestor species of *A. nanus*, *M. truncatula*, *T. pratense*, and *L. albus* diverged into *L. albus*; *AnTLP24* and *AnTLP28* were lost when diverged into *M. truncatula*, and nine orthologous genes of *AnTLP* were lost when diverged into *T. pratense*. Nine *AnTLP* were formed by means of segmental duplication, when the common ancestor species of *A. nanus*, *M. truncatula*, *T. pratense*, and *L. albus* diverged into *A. nanus*. It is worth noting that seven of these nine genes are formed by tandem duplication.

Gene duplication is considered to be one of the main driving forces of genome evolution. Segmental duplication, tandem duplication, and transposition events are the three main modes of gene expansion. Among these modes, fragment duplication and tandem duplication are considered to be the two main drivers of gene family expansion in plants [31]. In the TLP gene family of *A. nanus*, there were 16 segmental duplication genes and 8 tandem duplication genes. Therefore, the TLP gene family of *A. nanus* was mainly formed by segmental duplication. It is worth noting that seven tandem repeat genes are located on chromosome 3, and five tandem repeat genes are closely arranged. According to the evolutionary relationship of *AnTLPs* reconstructed in this study, *AnTLP12* may appear in the ancestral species of *A. thaliana* and grape, while *AnTLP13*, *AnTLP14*, *AnTLP15*, and *AnTLP16* may be generated by gene duplication during differentiation into *A. nanus*. It is speculated that *AnTLP13*, *AnTLP14*, *AnTLP15*, and *AnTLP16* may be formed by tandem duplication of *AnTLP12*. Therefore, the similarities and differences of these five *AnTLPs* in structure and function may have research value.

Ka/Ks value can be used to reflect the evolutionary direction of genes in environmental selection. A Ka/Ks value greater than 1 indicates that the gene is positive selection, Ka/Ks less than 1 indicates that the gene is purifying selection, and Ka/Ks equal to 1 indicates that the gene evolution is not affected by environmental pressure [32,33,34]. The Ka/Ks of all duplicated TLP genes in this study were less than 1, which means that all duplicated genes have undergone purifying selection in the environment. The Ka/Ks values of *A. nanus* duplicated genes were between 0.093 and 0.422, and the Ka/Ks values of some duplicated genes were higher, indicating that these genes were under strong environmental selection pressure. Codon usage bias can reflect the type of pressure on gene evolution [35,36]. In the present study, most of the points in the association map of ENC and GC3s were distributed near the standard curve, indicating that the evolution of TLP genes in *A. nanus* was affected by environmental selection pressure. We also noticed that there was a certain correlation between GC12 and GC3s of the TLP gene family, indicating that the evolution of the TLP gene in *A. nanus* was also affected by mutation pressure.

The cis-acting elements in the promoter region play important roles in the regulation of gene expression [37,38,39]. There was a large number of cis-acting elements related to plant response to abiotic stress signal predicted from promoters of *AnTLP*s, and these cis-acting elements included ABRE, LTR, and MBS. There were cis-acting elements related to low-temperature stress response in the promoter region of 17 *AnTLP* genes, such as *AnTLP12* and *AnTLP13*. There were cis-acting elements associated with drought stress response in the promoter region of 15 *AnTLP* genes, such as *AnTLP10* and *AnTLP28*. Analysis of transcriptome data showed that *AnTLP12* and *AnTLP13* were significantly up-regulated under low-temperature stress, which was in line with the results of cis-acting elements prediction of *AnTLP12* and *AnTLP13*. *AnTLP13* was transformed into *E. coli*, yeast, and tobacco to investigate the biological function of *AnTLP13* in low-temperature stress tolerance. Compared with the control, *E. coli* and yeast overexpressing *AnTLP13* gene showed stronger tolerance to low temperature. When the *AnTLP13* gene was transiently expressed in tobacco leaves, the transgenic tobacco had stronger freezing tolerance than the control. These results demonstrated that *AnTLP* genes such as *AnTLP13* probably contribute to the high tolerance to low temperature in *A. nanus*. Indeed, TLP from other plant species has been proved to be involved in low-temperature adaptation. For example, overexpression of the wheat *TaTLP2* gene in yeast can alleviate the damage of yeast in cold, heat, osmotic, and salt stress [9].

## 4. Materials and Methods

### 4.1. Identification of the TLP Proteins in A. nanus

The genomic sequences and annotation information of *A. nanus* were downloaded from GigaScience Database (http://gigadb.org/, accessed on 16 August 2021, accession number 100466) [40,41]. The genomes of *A. thaliana*, *V. vinifera*, *M. truncatula*, *T. pratense*, and *L. albus* were downloaded from the Phytozome 13 database (https://phytozome-next.jgi.doe.gov/, accessed on 16 August 2021, Phytozome genome ID: 167, 457, 385, and 567). HMMER3 software was used to identify the TLP family members in *A. nanus* [42], based on the TLP domain model (PF01167) in the Pfam database. All candidate sequences were manually checked using the HMMER web server (https://www.ebi.ac.uk/Tools/hmmer/, accessed on 16 August 2021). The physicochemical properties of TLP were predicted using the ProtParam tool (http://web.expasy.org/protparam/, accessed on 30 August 2021) [43]. Subcellular localization predictions were conducted using the WoLF PSORT tool (http://www.genscript.com/psort/wolf_psort.html, accessed on 30 August 2021) [44].

### 4.2. Chromosomal Location and Gene Structure Analysis of A. nanus TLP Family Genes

Based on annotation information from the *A. nanus* genome, the location of AnTLP on chromosomes and the exon–intron distribution of AnTLP were visualized using TBtools software [45]. MEME (Multiple Expectation Maximization for Motif Elicitation) (http://meme-suite.org/, accessed on 30 August 2021) [46] was used to identify the conserved motif with a minimum width of 6, a maximum width of 50, a motif number of 20, and E-value < 0.05.

### 4.3. Multiple Sequence Alignment and Phylogenetic Analysis

Multiple sequence alignment was performed using the MUCSLE algorithm [47]. The phylogenetic tree was constructed using the MEGA X [48], and bootstrap analysis was conducted using 1000 replicates. The synteny analyses of AnTLP were performed using the MCScanx tools [49]. The phylogenetic tree was embellished using Evolview (https://www.evolgenius.info/evolview/, accessed on 30 August 2021) [50]. The synonymous substitution rate (Ks), nonsynonymous substitution rate (Ka), and Ka/Ks ratio between homologous gene pairs were calculated using KaKs_Calculator 2.0 [51]. The time of divergence between the two species was queried through the TIMETREE website (http://www.timetree.org/, accessed on 30 August 2021) [52]. The duplication time of homologous genes within the *A. nanus* gene family was calculated using the formula T = Ks/2λ. Codon bias analysis of the TLP gene family was performed using CodonW software.

### 4.4. Prediction of Cis-Acting Elements in Promoter Regions of TLP Genes

PlantCARE database(https://bioinformatics.psb.ugent.be/webtools/plantcare/html/, accessed on 26 January 2022) [53] was used to predict the cis-acting elements in the 2000 bp promoter region upstream of each TLP gene’s start codon, and the results were visualized using TBtools software [45].

### 4.5. Gene Expression Analysis Based on the Transcriptome Data

A total of 12 transcriptomic datasets of *A. nanus* were downloaded from the SRA database with accession numbers SRR11089024–SRR11089029 and SRR11087599–SRR11087604, which contained transcriptome data from the control group, osmotic treatment group (20% PEG-6000 solution for 7 days), cold-stress treatment group (4 °C for 7 days), and *A. nanus* leaves in spring and winter, respectively. The gene expression level of each gene was calculated using the Kallisto quant [54].

### 4.6. Plant Materials and Stress Treatment

The seeds of *A. nanus* were collected from Wuqia county, Xinjiang autonomous district, China. The seed germination and planting conditions of *A. nanus* were based on a previous study [55]. Osmotic and cold-stress treatments were performed with reference to a previous study [24]. In brief, seedlings of *A. nanus* were randomly divided into 9 groups, and one group grew at normal conditions and was used as the control group. The four osmotic stress treatment groups were irrigated with 20% PEG-6000 for 3 h, 6 h, 12 h, and 24 h. The other four groups were transferred to a 4 °C incubator for cold-stress treatment for 3 h, 6 h, 12 h, or 24 h. Leaf samples from the control groups and the treatments group were collected and snap-frozen in liquid nitrogen, then the samples were stored at −80 °C until RNA extraction.

### 4.7. RNA Extraction and Quantitative Real-Time PCR (qRT-PCR) Analysis

Total RNA was extracted from the leaves of *A. nanus* using the Trizol reagent following the manufacturer’s directions (Invitrogen, Carlsbad, CA, USA), and reverse transcription was conducted using a FastQuant RT Kit (with gDNase) (TIANGEN, Beijing, China). The qRT-PCR analysis was performed according to the methods described previously [56], and the eukaryotic translation initiation factor 1 (*eIF1*) gene was used as the internal control. The primers used for qRT-PCR are listed in Supplementary Table S3. Three biological replicates were used for each group, and three technical replicates of each biological replicate were analyzed. The relative expression of the genes was calculated using the 2^−ΔΔCt^ method [57].

### 4.8. Vector Construction and Expression in E. coli

The AnTLP13 was expressed in *E. coli* by reference to the previous method [58]. The vector used in the experiment was pET-28a(+), the enzyme digestion sites were *BamH*I and *Hind*III, and the competent cell was *E. coli* BL21(DE3). The normal growth temperature of *E. coli* is 37 °C, and the temperature was lowered to 28 °C to simulate cold-stress treatment. When the *E. coli* was cultured under cold stress, the OD600 value of the medium was recorded every 1 h. When *E. coli* was in the logarithmic phase, the culture medium was transferred to a −20 refrigerator for freezing treatment. *E. coli* was frozen at −20 °C for 1 h and then thawed at room temperature, and the treatment was repeated three times. Finally, the survival rate of *E. coli* after freezing treatment was calculated by colony forming unit (CFU) on LB solid medium. Three independent biological replicates were performed.

### 4.9. Vector Construction and Expression in Yeast

The *AnTLP13* gene was expressed in yeast according to a previous method [59]. *AnTLP13* gene was inserted into the pYES2 vector by the restriction sites of *Hind*Ⅲ and *Bam*HI, and then the recombinant plasmid was transferred into the INVScl yeast strain. When the yeast was in the logarithmic phase, the culture medium was transferred to −20 refrigerator for freezing treatment. Yeast was frozen at −20 °C for 1 h and then thawed at room temperature, and the treatment was repeated three times. Finally, the effect of *AnTLP13* on yeast freezing tolerance was analyzed by comparing the number of colonies on SD-Ura solid medium.

### 4.10. Transient Transformation of AnTLP13 in Tobacco

Seeds of *Nicotiana benthamiana* were sown in peat soil and vermiculite matrix at a fully mixed volume ratio of 1:1. The seedlings were cultured in a greenhouse at 25 °C, with a light intensity of 400 μmol·m^−2^·s^−1^ and a photoperiod of 16/8 h (light/dark). Six seedlings with similar growth status were selected and divided into two groups. The AnTLP13 was transiently expressed in tobacco by reference to the previous method [60]. The vector used in the experiment was pCAMBIA1305, the enzyme digestion sites were *Xba*I and *BamH*I, and the competent cell was *Agrobacterium tumefaciens* GV3101. One group of tobacco plants was transformed transiently with pCAMBIA1305 empty vector, and the other group of plants was transformed transiently with pCAMBIA1305 vector ligated with *AnTLP13*. All tobacco plants were transferred to a −4 °C plant incubator for cold-stress treatment for 12 h. After freezing-stress treatment, the growth state of tobacco was observed. MDA and REL were measured according to a previously described method [61]. Three biological replicates were executed in the transient expression experiments. The subcellular locations of the AnTLP13 were imaged using an OLYMPUS Inverted Fluorescence Microscope IX81.

### 4.11. Statistical Analysis

Determination of the physiological indexes was performed in six replicates. All data were calculated using Microsoft Excel 2019 for mean and standard deviation. Analysis of variance (Duncan’s) was performed using R software, and ‘*’ and ‘**’ indicated *p* < 0.05 and *p* < 0.01, respectively.

## Figures and Tables

**Table 1:** Characterization of the predicted TLPs in *A. nanus.*

Sequence ID	Gene Name	Genome Location	Amino Acid Length	Molecular Weight/kD	Theoretical pI	GRAVY	Signal Peptide	TMHs	Subcellular Localization *
EVM0028587	AnTLP1	chr1: 84629737-84630810	237	25.36	7.43	0.157	1-23	0	extr
EVM0000288	AnTLP2	chr2: 3943782-3944871	245	26.88	6.76	−0.371	1-25	0	extr
EVM0012162	AnTLP3	chr2: 3949219-3950299	241	26.33	5.33	−0.068	1-39	1	extr
EVM0031681	AnTLP4	chr2: 11425283-11428175	304	31.34	4.45	0.062	1-26	1	chlo
EVM0023773	AnTLP5	chr2: 11445664-11447849	316	32.84	5.22	−0.075	1-28	1	extr
EVM0003940	AnTLP6	chr2: 77169927-77172678	313	32.80	6.19	−0.147	1-35	0	extr
EVM0027347	AnTLP7	chr2: 77240207-77241420	196	20.87	5.00	0.343	1-23	1	extr
EVM0011680	AnTLP8	chr2: 91149625-91150307	212	22.51	5.85	0.31	1-24	1	golg
EVM0028885	AnTLP9	chr2: 97035730-97037285	250	27.01	9.25	0.037	1-28	1	extr
EVM0036902	AnTLP10	chr3: 3630861-3632795	272	29.75	6.89	−0.059	1-36	1	extr
EVM0012276	AnTLP11	chr3: 3636405-3640791	225	24.81	8.57	−0.145	NO	1	extr
EVM0027709	AnTLP12	chr3: 60728536-60730716	242	25.54	4.78	−0.167	1-21	0	extr
EVM0030154	AnTLP13	chr3: 60822641-60824723	242	25.62	4.78	−0.236	1-21	0	extr
EVM0006019	AnTLP14	chr3: 60829095-60830741	240	26.04	4.97	−0.138	1-22	0	extr
EVM0035676	AnTLP15	chr3: 60870728-60872244	232	25.08	4.92	−0.074	NO	0	extr
EVM0033132	AnTLP16	chr3: 60875649-60877597	270	29.52	4.7	−0.25	NO	0	chlo
EVM0026680	AnTLP17	chr3: 66587898-66589488	228	24.26	4.37	−0.196	NO	0	extr
EVM0006621	AnTLP18	chr4: 5755011-5756575	249	26.13	7.38	0.105	1-26	1	extr
EVM0005233	AnTLP19	chr4: 62429388-62432876	300	31.55	5.39	−0.168	NO	1	extr
EVM0005658	AnTLP20	chr4: 81197773-81199137	257	27.41	7.17	−0.023	1-31	1	chlo
EVM0036386	AnTLP21	chr4: 85077259-85079741	348	35.13	4.24	0.02	1-22	1	extr
EVM0034723	AnTLP22	chr4: 85093701-85097072	315	33.52	8.75	0.151	1-24	0	extr
EVM0007971	AnTLP23	chr5: 8628389-8630328	245	25.71	6.07	0.164	1-22	0	extr
EVM0028324	AnTLP24	chr5: 12202458-12204306	307	32.80	5.02	−0.006	1-25	2	extr
EVM0001585	AnTLP25	chr5: 14996048-14997745	287	30.79	8.65	0.08	1-27	1	plas
EVM0014534	AnTLP26	chr6: 3060387-3064918	246	26.66	4.87	−0.243	1-21	0	chlo
EVM0005703	AnTLP27	chr6: 4178850-4180284	240	24.7	5.37	−0.075	NO	0	chlo
EVM0035081	AnTLP28	chr6: 19546129-19547818	309	33.29	9.02	−0.002	1-27	2	extr
EVM0034418	AnTLP29	chr7: 75592532-75595722	325	34.15	4.82	0.051	1-22	0	extr
EVM0006518	AnTLP30	chr8: 57726394-57727987	288	31.30	6.29	−0.111	1-27	0	extr
EVM0016538	AnTLP31	chr9: 56159846-56162293	264	28.23	5.48	−0.059	NO	0	extr

* extr: extracellular matrix, chlo: chloroplast, plas: plasm membrane, golg: golgi apparatus.

**Table 2:** Analysis of evolutionary selection pressure on the *A. nanus* TLP gene family.

Segment Pairs	Ka	Ks	Ka/Ks	T(MYA)	Selection Pressure
*AnTLP1-AnTLP18*	0.203	2.191	0.093	534.39	Purifying selection
*AnTLP4-AnTLP22*	0.381	1.170	0.325	285.37	Purifying selection
*AnTLP5-AnTLP21*	0.274	1.805	0.152	440.24	Purifying selection
*AnTLP6-AnTLP21*	0.266	1.202	0.221	293.17	Purifying selection
*AnTLP6-AnTLP5*	0.152	0.724	0.210	176.59	Purifying selection
*AnTLP7-AnTLP22*	0.491	1.521	0.323	370.98	Purifying selection
*AnTLP7-AnTLP4*	0.325	0.827	0.392	201.71	Purifying selection
*AnTLP8-AnTLP2*	0.463	3.322	0.139	810.24	Purifying selection
*AnTLP23-AnTLP1*	0.058	0.402	0.144	98.05	Purifying selection
*AnTLP24-AnTLP28*	0.139	0.331	0.422	80.73	Purifying selection
*AnTLP26-AnTLP29*	0.187	0.827	0.227	201.71	Purifying selection
*AnTLP26-AnTLP4*	0.274	1.313	0.209	320.24	Purifying selection
*AnTLP29-AnTLP4*	0.290	2.994	0.097	730.24	Purifying selection

**Table 3:** Analysis of the codon usage bias of the *A. nanus* TLP gene family.

Gene Name	CAI	Fop	ENC	Gene Name	CAI	Fop	ENC
*AnTLP25*	0.19	0.37	50.35	*AnTLP9*	0.24	0.48	44.78
*AnTLP24*	0.21	0.37	50.83	*AnTLP31*	0.25	0.42	53.03
*AnTLP23*	0.23	0.38	52.40	*AnTLP20*	0.30	0.55	46.70
*AnTLP30*	0.19	0.36	50.18	*AnTLP19*	0.20	0.44	55.75
*AnTLP1*	0.20	0.30	48.39	*AnTLP21*	0.20	0.44	55.19
*AnTLP28*	0.17	0.35	53.65	*AnTLP22*	0.25	0.50	56.81
*AnTLP26*	0.31	0.55	57.45	*AnTLP18*	0.18	0.34	47.94
*AnTLP27*	0.29	0.55	56.25	*AnTLP14*	0.22	0.35	49.64
*AnTLP29*	0.26	0.46	60.52	*AnTLP10*	0.24	0.38	48.33
*AnTLP3*	0.29	0.54	54.28	*AnTLP16*	0.24	0.39	57.74
*AnTLP8*	0.24	0.47	61.00	*AnTLP11*	0.22	0.42	54.54
*AnTLP6*	0.23	0.46	60.14	*AnTLP15*	0.23	0.37	48.21
*AnTLP2*	0.27	0.52	56.00	*AnTLP12*	0.27	0.46	60.68
*AnTLP5*	0.23	0.45	58.46	*AnTLP13*	0.28	0.45	59.12
*AnTLP7*	0.21	0.43	51.37	*AnTLP17*	0.28	0.46	55.33
*AnTLP4*	0.25	0.48	51.17				

## Data Availability

No new data were created or analyzed in this study. Data sharing is not applicable to this article.

## Supplementary Materials

The following supporting information can be downloaded at: https://www.mdpi.com/article/10.3390/ijms24032209/s1.
